# Editorial: Food Protein-Based Colloids: Structure, Digestion, and Nutrients Delivery

**DOI:** 10.3389/fnut.2022.982366

**Published:** 2022-07-18

**Authors:** Yuan Li, Weilin Liu, Pete Wilde, Jianhua Liu

**Affiliations:** ^1^Key Laboratory of Precision Nutrition and Food Quality, Key Laboratory of Functional Dairy, College of Food Science and Nutritional Engineering, China Agricultural University, Beijing, China; ^2^School of Food Science and Biotechnology, Zhejiang Gongshang University, Hangzhou, China; ^3^Quadram Institute Bioscience, Norwich Research Park, Norwich, United Kingdom; ^4^College of Food Science and Technology, Zhejiang University of Technology, Hangzhou, China

**Keywords:** protein-based colloids, emulsion, gel, digestion, structure

## Introduction

There has been much interest over the years in protein-based colloidal systems and utilizing the functionality of protein in food systems has been a target for many researchers and there is a huge literature on their gelling, thickening, emulsifying, and foaming properties. Proteins are seen as a natural alternative to synthetic emulsifiers and stabilizers, which are generally perceived to be artificial and associated with unhealthy ultra-processed foods. Therefore, this earlier research on protein functionality is now being exploited to formulate protein-based colloidal structures with functionalities capable of delivering health benefits. The papers in this special issue reflect very well the current and emerging trends in protein-colloid research in the food and nutrition space.

## Protein-based particles

Powder and particulate technology have been used to develop powdered or granulated systems with a low water content, and hence long shelf life and low transport costs, but could be reconstituted easily at point of use. By creating the right structure with the right properties, such colloidal particles can be loaded with bioactive compounds to improve storage stability and functionality. Zhang et al. showed that they could create particles using the maize protein zein complexed with gum Arabic. The particle stability was improved by adding tannic acid to enhance the intermolecular interactions. Similarly, Chen et al. complexed zein with glycosylated lactoferrin to form nanoparticles that could encapsulate the flavone 7,8-dihydroxyflavone (7,8-DHF), which had good stability, high encapsulation efficiency and *in vitro* bioaccessibility of the DHF.

## Simple emulsion systems

Proteins can stabilize emulsions, but they are not always as effective as small molecule emulsifiers, but they can add other functionalities to an emulsion. The first example is from Cai et al. where they tried to enhance the functionality of thymol, a natural antibiotic by encapsulating it with lauric acid in a caseinate stabilized emulsion. The caseinate emulsions are highly stable, but readily digestible, making the thymol available to control bacterial growth. Wang Q. et al. found that certain ratios of whey protein and caseinate could stabilize the emulsion against calcium induced aggregation and also improve oxidative stability, potentially offering a solution to improve the nutritional and functional properties of infant formulae.

## Pickering emulsions

Pickering emulsions are stabilized by particles which are held at the interface by surface interactions between the three phases (oil, water, and particle). Their application in food systems has been limited, and the most effective Pickering particles are synthetic and not suitable for human consumption. More recent work has focussed on the use of food grade particles, such as protein aggregates to form food-suitable Pickering emulsions. Ren et al. utilized insoluble protein nano-particles from tea residues as natural, sustainable emulsifiers. The emulsions showed tuneable rheological properties as a function of ionic strength. The high stability of Pickering emulsions is further demonstrated by Shen et al. who combined bacterial nano-cellulose and soy protein to create strong, stable particles through anti-solvent precipitation. These particles offered excellent emulsion stability, and improved the bioavailability of curcumin. A similar approach was used by Kiat Wong et al. who also used cellulose and soy protein to form Pickering emulsions which were incorporated into gel beads as a targeted delivery device to control release of the encapsulated bioactive compound. Finally, Wang J. et al. used whey protein aggregates to stabilize emulsions containing the long chain fatty acid DHA. This approach again improved the oxidative stability and delivery of the DHA.

## Emulsion gels

Emulsion gels are an interesting composite system as their rheological and functional properties depend on both the emulsion and the gel matrix as well as the interactions between the phases. These areas have been reviewed by Abdullah et al. showing how research on the gel-matrix interactions is being applied to functionalities such as fat replacement, controlled release and probiotic delivery, demonstrating the versatility of these structures to provide multiple functionalities in food products. This was demonstrated by Su et al. who found that high pressure processing could influence interactions between the emulsion and gel phases, altering not only the gel properties but also the delivery of encapsulated curcumin. Similarly, as discussed earlier, the incorporation of Pickering emulsions into gel beads could also be used to control release of bioactives (Kiat Wong et al.).

## Emulsifier replacement

There is a drive toward replacing artificial emulsifiers with more natural alternatives. Protein-particles can create very stable Pickering emulsions, but tend to have large droplet size, which is not always desirable in food systems. To address this, the surface activity and structure of the protein molecule itself needs to be altered. Li et al. have reviewed a specific approach of utilizing protein-polyphenol complexes to alter the secondary structure, hydrophobicity and flexibility of the protein. This can significantly improve the emulsifying properties of the protein, leading to finer, more stable emulsions, acting as possible replacements of artificial emulsifiers, whilst also adding further functionalities such as improved oxidative stability and delivery of bioactive compounds.

## Plant based systems

Finally, another global food challenge is the move away from animal protein toward plant-based systems. Most plant proteins have poor solubility and require extensive processing to improve functionality. Some of the papers already described are addressing this challenge, to improve the functional properties of plant proteins. All these papers are effectively utilizing the poor solubility of plant proteins, to form particles to either act as functional carriers themselves (Zhang et al.) or to stabilize Pickering emulsions with plant protein-based particles (Kiat Wong et al.; Ren et al.; Shen et al.).

## Summary

The utilization of protein-based colloidal systems has been based on many years of fundamental research into the molecular, colloidal, structural, and functional properties of these systems. This research is currently being applied to develop intelligently designed structures with enhanced functionalities, particularly the oxidative stability and bioavailability of beneficial bioactive compounds whilst also addressing the demand for more natural and sustainable food ingredients.

## Author contributions

YL, WL, and JL: writing—review and editing. PW: writing—original draft. All authors contributed to the article and approved the submitted version.

## Funding

PW gratefully acknowledges the support of the Biotechnology and Biological Sciences Research Council (BBSRC) through the BBSRC Institute Strategic Programme Food Innovation and Health BB/R012512/1.

## Conflict of interest

The authors declare that the research was conducted in the absence of any commercial or financial relationships that could be construed as a potential conflict of interest.

## Publisher's note

All claims expressed in this article are solely those of the authors and do not necessarily represent those of their affiliated organizations, or those of the publisher, the editors and the reviewers. Any product that may be evaluated in this article, or claim that may be made by its manufacturer, is not guaranteed or endorsed by the publisher.

